# AI-based thematic exploration to understand patients with myasthenia gravis by serological subtype

**DOI:** 10.3389/fneur.2025.1576405

**Published:** 2025-07-28

**Authors:** Louis Jackson, Caroline Brethenoux, Alyssa DeLuca, Jacqueline Pesa, Zia Choudhry, Patrick Furey, Rosario Alvarez, Laura Gonzalez, Alex Lorenzo, Raghav Govindarajan, Ashley E. L. Anderson

**Affiliations:** ^1^Johnson & Johnson, Horsham, PA, United States; ^2^Human Dot Plus, Dallas, TX, United States; ^3^HSHS Medical Group Multispecialty Care – St. Elizabeth’s, O’Fallon, IL, United States; ^4^Houston Methodist, Neuromuscular Division, Houston, TX, United States

**Keywords:** myasthenia gravis, digital conversations, patient perspective, serostatus, sentiment analysis

## Abstract

**Objective:**

Myasthenia gravis (MG) is challenging to diagnose and appropriate treatment is informed by serological versus diagnostic testing. Digital conversations can reveal insights into patient perceptions and concerns that may differ across autoantibody subtypes. The purpose of this study was to describe MG-related sentiments, barriers, and drivers by describing digital conversations by serostatus/serotype.

**Methods:**

US-based public-domain conversations focusing on MG and posted within topical sites, message boards, social networks, and blogs from August 2022 to August 2023 were mined. Content contributors self-identified by MG serostatus (seropositive/seronegative/AChR+/MuSK+/LRP4+) within the conversations or on public profiles. Advanced search techniques and AI-powered algorithms were used to extract/organize data by topics into a large, unstructured dataset. Natural language processing identified frequent topics, sentiments, mindsets, and drivers/barriers to treatment.

**Results:**

In total, 8,784 conversations were mined from seropositive patients and/or their caregivers (including 1,058 AChR+, 589 MuSK+, and 537 LRP4+) and 2,261 were mined from seronegative patients and/or their caregivers. All conversations revealed an uncertain mindset, with a struggling mindset more prevalent among seronegative posts and ~40% of seropositive posts deemed utilitarian/indomitable. Positive drivers of sentiment were not seen for any subgroup. Negative drivers centered on misdiagnosis and symptoms more frequently in seronegative posts, and on impact on life in seropositive posts. Instability of symptom control in all subgroups impacted quality of life.

**Conclusion:**

Conversations revealed the burden of misdiagnosis for the seronegative group, negative impact on life for the seropositive group, and overall difficulty of the MG diagnostic journey. Digital conversations across MG serostatus subgroups related to the patient/caregiver experience of living with MG might provide useful insights for healthcare professionals seeking to improve patient care.

## Introduction

Myasthenia gravis (MG) is a rare autoantibody disorder that affects the neuromuscular junction, resulting in fluctuating muscle weakness and susceptibility to fatigue (1). Serological testing is used to identify the presence of autoantibodies targeting the acetylcholine receptor (AChR+; ~85% of cases), muscle-specific receptor tyrosine kinase (MuSK+; ~6% of cases), and low-density receptor-related protein 4 (LRP4+; ~2% of cases) (2–4). Improvements in methods for detection, including cell-based assays, have enabled the detection of AChR antibodies that are undetectable by conventional methods (5, 6). When blood tests fail to identify any of these antibodies, the patient is considered seronegative (~10%) (3).

The clinical significance of subtype differences has been shown in severity of disease, number and range of symptoms, frequency of MG exacerbations, and treatment side effects. AChR antibodies are mainly of the immunoglobulin G1 (IgG1) and IgG3 subclasses, while MuSK antibodies are primarily of the IgG4 subclass (3). In patients with MuSK+ MG, muscle weakness mainly affects cranial and bulbar muscles, leading to neck and respiratory symptoms (3). MuSK+ MG is more prevalent in young adults and people of African or equatorial descent (3). Patients with LRP4 + MG tend to present before 50 years of age and have generally milder symptoms than other MG subtypes (3). Long-established therapies for MG include pyridostigmine and non-specific immune therapies such as corticosteroids and non-steroidal immunosuppressants (7). More recently, targeted therapies such as rituximab, eculizumab (a terminal C5 complement inhibitor), and neonatal Fc receptor (FcRn) antagonists such as efgartigimod have been introduced (8–10). Patients with MuSK+ MG respond poorly to pyridostigmine and eculizumab is only indicated in AChR+ MG (7, 8), although the recently approved FcRn rozanolixizumab-noli is indicated for both MuSK+ and AChR+ subtypes (10). Compared with seropositive patients, seronegative patients report more symptoms, more frequent exacerbations, more severe treatment side effects, greater quality-of-life limitations, greater impact of treatment on daily life, and more burdensome diagnostic challenges (11, 12). Furthermore, no modern treatments are FDA-approved for use in seronegative patients (12).

With serological status disproportionately weighted toward AChR+, there is a lack of information around patient and caregiver experiences and concerns across the less common MG subtypes. Digital conversation data can reveal insights into patient/caregiver perceptions and concerns and provide a unique opportunity to gain insight into their perspectives based on serological status.

The purpose of this study was to explore digital conversations describing MG-related sentiments, mindsets, barriers, and drivers by MG serostatus/serotype.

## Materials and methods

The analysis was based on US-based public domain patient and/or caregiver conversations focusing on MG and posted from August 2022 to August 2023. Digital conversations were limited to those originating from US IP addresses. Human Dot Plus (HDP, previously CulturIntel™) employed its proprietary AI-powered methodology to mine, structure, and analyze unsolicited public domain digital conversations using advanced Natural Language Processing (NLP) and text analytics, as previously described (13). Data were collected from a wide range of online sources including forums, blogs, message boards, and social media platforms, focusing exclusively on open-source, user-generated content. All posts were counted only once and any duplicate posts were filtered out using the highly effective GPU-Accelerated Deduplication protocol (14). No personally identifiable information was collected; all data were anonymized and aggregated and never tracked to individual users. Ethics approval was not required. The study adhered to Article 32 of the General Data Protection Regulations.

HDP uses “internet-wide web scraping” to extract first-party public content across the web. Conversations were tagged based on self-identification (e.g., “I am a patient”) as detected in user posts or public profiles and no further methodology to validate user status was applied. Caregiver posts were included. MG serostatus (seropositive, seronegative) and subtype (AChR+, MuSK+, LRP4+) were also self-identified. NLP and AI algorithms were trained and validated to classify sentiment (positive, negative, and neutral), identify recurring themes, and detect drivers and barriers to health behaviors. No themes were created in advance – categories were derived from naturally occurring patterns within the data.

Digital conversations were sorted and analyzed thematically by serostatus and subtype group. Thematic analysis was conducted through a human-assisted AI workflow with iterative training and validation. A “Hold-out” cross-validation protocol was used: 80% of the dataset served as the training set and 20% as the test set, with models trained to a ≥ 99% accuracy threshold. Large Language Models (LLMs) powered the classification and interpretation processes. These models were pre-trained and subsequently fine-tuned for task-specific objectives. To minimize bias and ensure consistency, all input data were treated equally, and categorizations were objectively produced through HDP’s established training, testing, and validation protocols.

Themes included the most frequently discussed topics emerging from the conversation data (categorized as diagnosis, treatment, living with MG, and symptoms), sentiments (positive, negative, and neutral factors), overarching mindsets toward MG (uncertain, utilitarian, struggling, indomitable), and drivers/barriers to treatment.

The model used a supervised learning approach for sentiment and mindset classification. A labeled dataset was created by manually annotating a corpus of patient/caregiver conversations with sentiment labels (positive, negative, neutral) and mindset categories (uncertain, utilitarian, struggling, indomitable). The labeled dataset was used to train the deep learning model to recognize patterns associated with different sentiments and mindsets. The k-fold technique was implemented for cross-validation to ensure that the model generalized well to new, unseen data. Model parameters were fine-tuned based on performance metrics to optimize accuracy (proportion of correctly classified instances across all sentiment and mindset categories), precision and recall (the model’s ability to avoid false positives and negatives for each category), and F1 score (the harmonic mean of precision and recall, providing a balanced measure of model performance). Confusion matrix analysis identified which sentiments or mindsets are most frequently misclassified to guide model improvements. The following techniques were used to refine and improve the analysis: active learning, whereby human feedback is incorporated to improve model performance on difficult or ambiguous cases; regular retraining, to update models with new data to capture evolving language patterns and emotional expressions in online discussions; and domain adaptation, whereby pre-trained models are adjusted to better fit the specific language and emotional contexts of patient and/or caregiver discussions.

Descriptive results are presented for seropositive and seronegative conversations, and within the seropositive conversations, those attributable to AChR+, MuSK+, and LRP4 + subgroups are presented.

## Results

**Figure 1:**
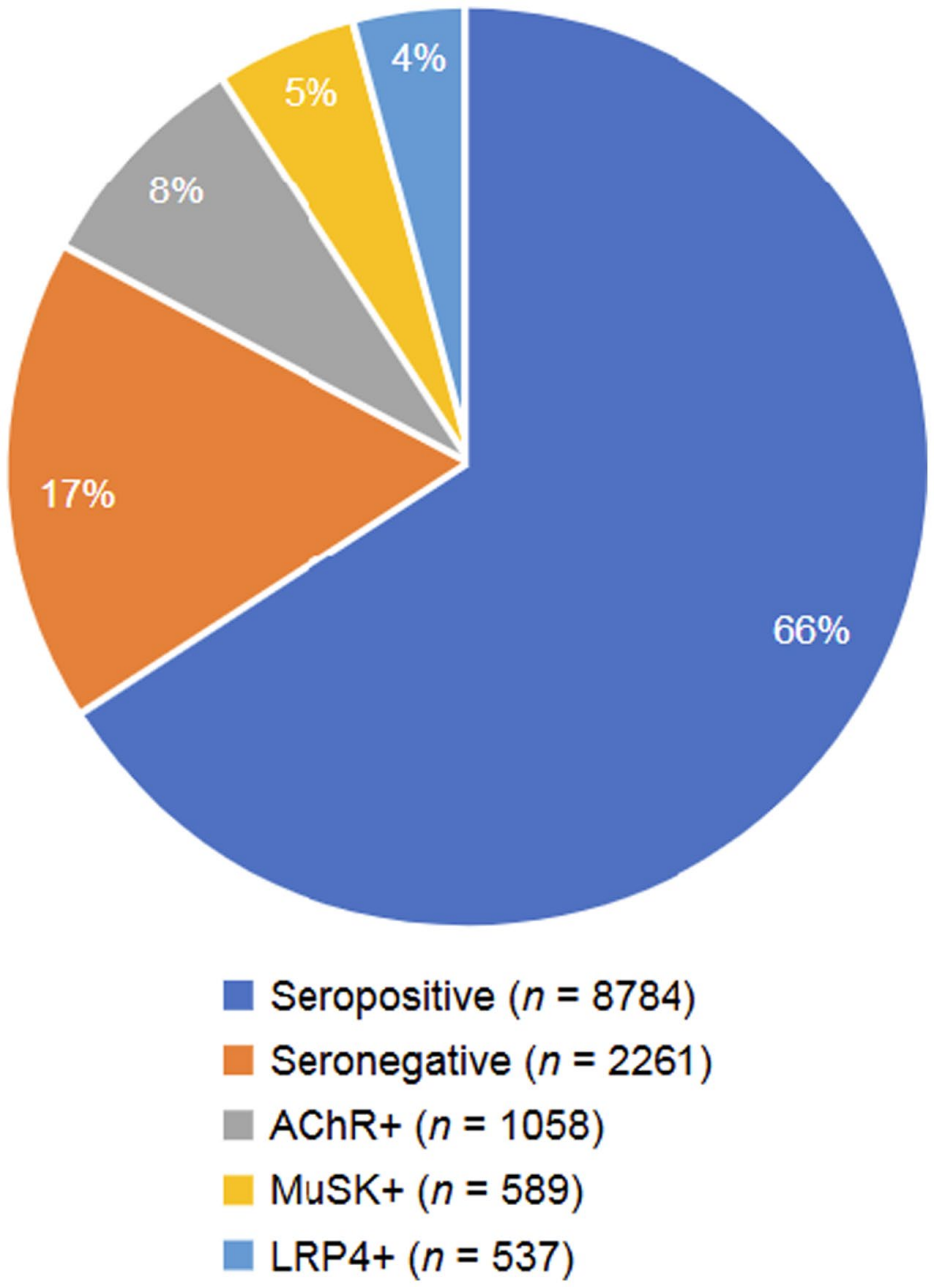
Digital conversations by MG serostatus. A total of 8,784 conversations were mined from the seropositive group (including 1,058 AChR+, 589 MuSK+, and 537 LRP4+) and 2,261 conversations were mined from the seronegative group. AChR, acetylcholine receptor; LRP4, low-density receptor-related protein 4; MuSK, muscle-specific receptor tyrosine kinase.

A total of 11,045 unique publicly available conversations focusing on MG were collected over a 12-month period from patients and/or caregivers. These included 8,784 conversations from the self-identified seropositive group and 2,261 from the self-identified seronegative group. Within the conversations by the seropositive subgroup, 25% (2,184 posts) further self-identified their serostatus; the AChR+ subgroup contributed 1,058 posts, the MuSK+ subgroup 589 posts, and the LRP4 + subgroup 537 posts (Figure 1). Message boards contributed 34% of the conversations, topical sites contributed 34%, social networks contributed 12%, blogs contributed 9%, content sharing contributed 7%, and comments contributed 4%.

### Patient sentiment

**Figure 2:**
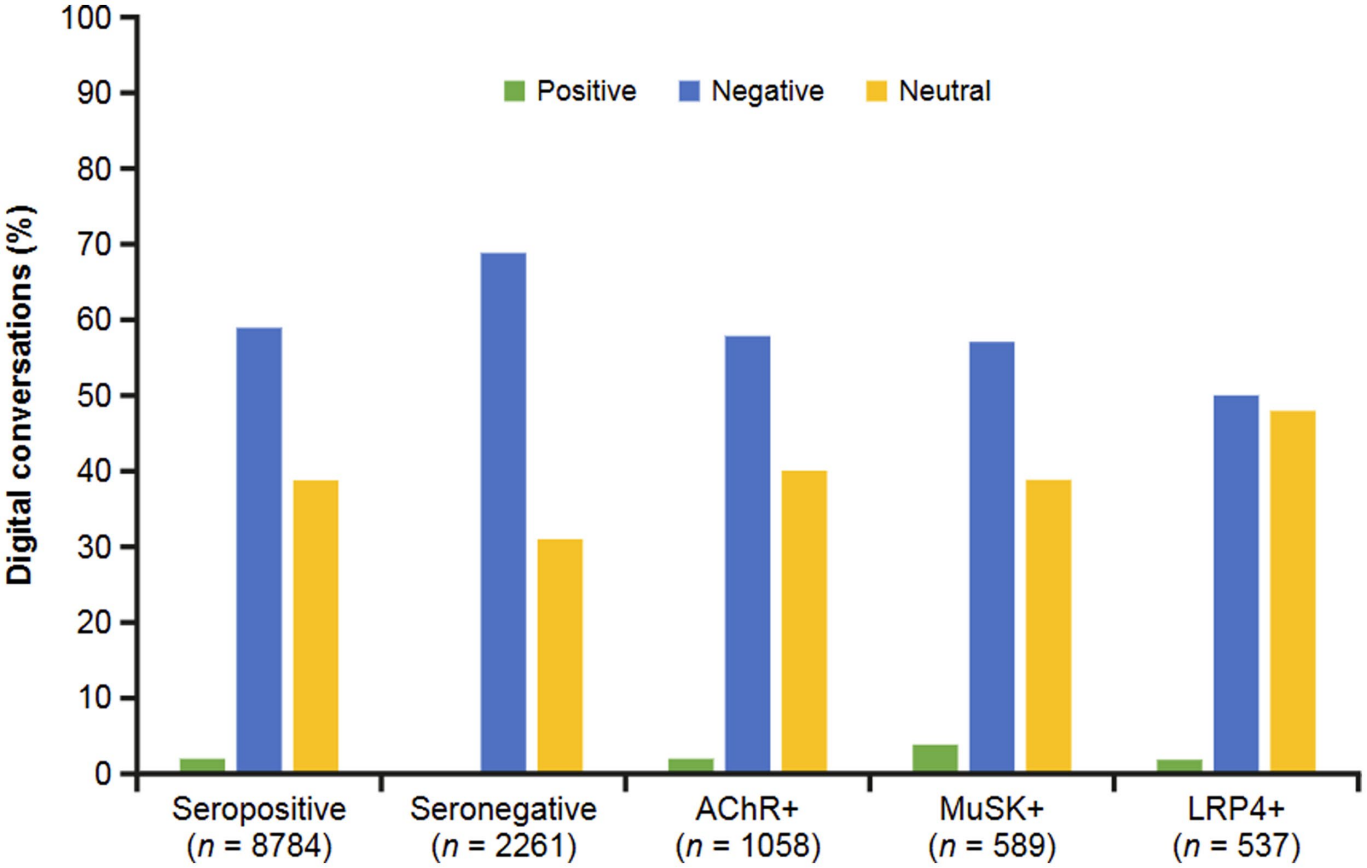
Sentiments by MG serostatus. Sentiments were classified as positive, negative, or neutral. Negative and neutral sentiments predominated in all groups. AChR, acetylcholine receptor; LRP4, low-density receptor-related protein 4; MuSK, muscle-specific receptor tyrosine kinase.

Regardless of antibody status, negative conversations were dominant (59% overall). The seronegative group had a higher percentage of negative conversations (69%), with the LRP4 + subgroup having the fewest (50%) (Figure 2). Positive conversations were rare, ranging from none in the seronegative group to 4% in the MuSK+ subgroup. For neutral conversations, the LRP4 + subgroup contributed at a higher frequency (48%) than the seropositive group and MuSK+ subgroup (39%), as well as the seronegative group (31%). Reasons for feeling negative, neutral, or positive about MG were identified and tabulated by subgroup.

**Figure 3:**
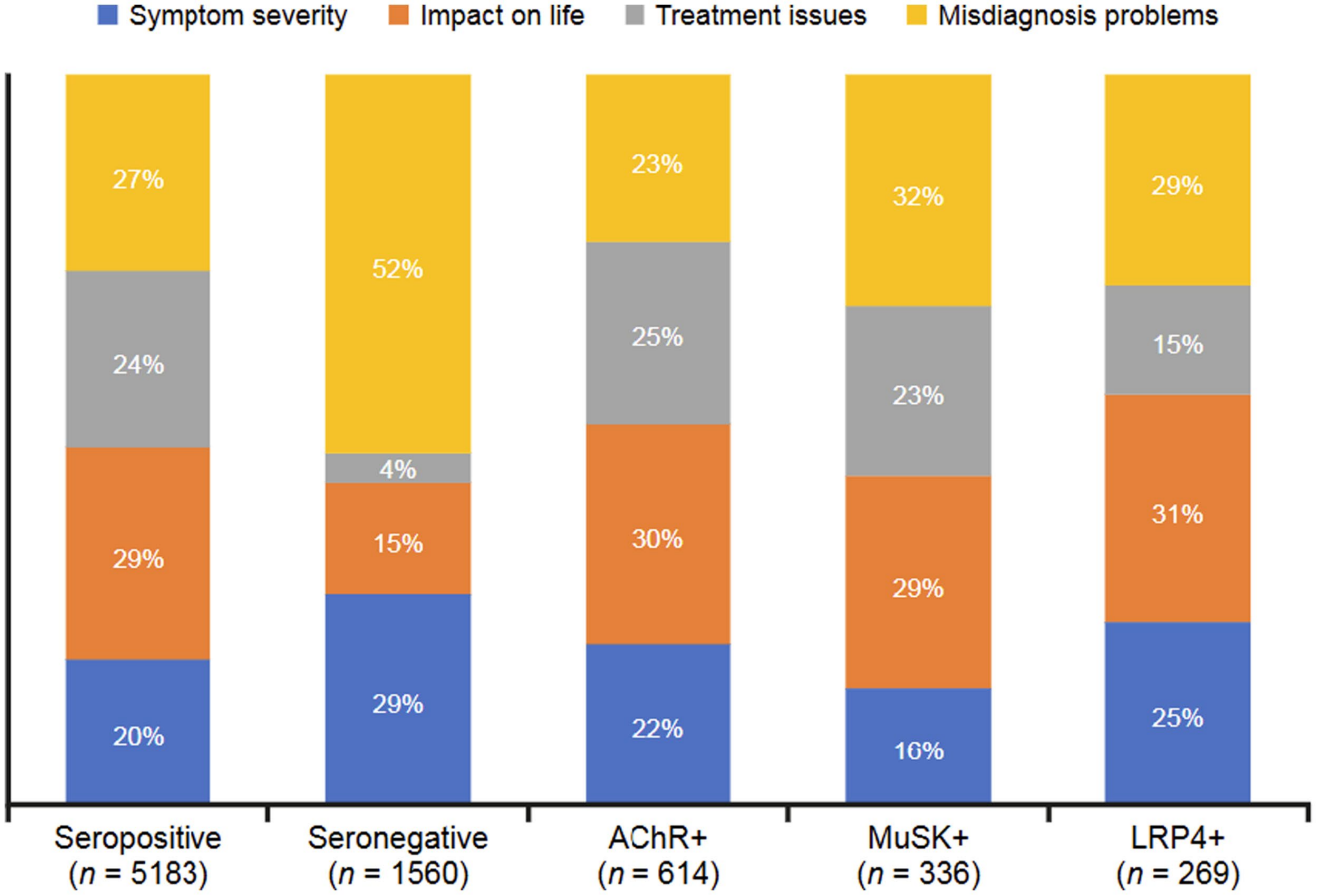
Negative drivers for sentiments expressed in digital conversations by serostatus. The dominant categories of reasons underlying negative sentiments were symptom severity (number of symptoms, uncomfortable physical experiences), impact on life (day-to-day tasks, quality of life), treatment issues (ineffective treatments, worsening symptoms), and misdiagnosis problems (wariness of HCPs, potential misdiagnosis). AChR, acetylcholine receptor; HCP, healthcare professional; LRP4, low-density receptor-related protein 4; MuSK, muscle-specific receptor tyrosine kinase.

The dominant categories of reasons patients and/or caregivers feel negatively toward MG (Figure 3) included symptom severity (such as number of symptoms, uncomfortable physical experiences), impact on life (day-to-day tasks, quality of life), treatment issues (ineffective treatments, worsening symptoms), and misdiagnosis problems (wariness of healthcare professionals [HCPs], potential misdiagnosis). Among the seronegative group, negative drivers centered on misdiagnosis problems (52%), followed by symptom severity (29%). Impact on life was the most frequent theme among the overall seropositive group (29%), with little variation among subgroups (AChR+, 30%; LRP4+, 31%; MuSK+, 29%). The MuSK+ subgroup frequently discussed misdiagnosis problems (32%).

**Figure 4:**
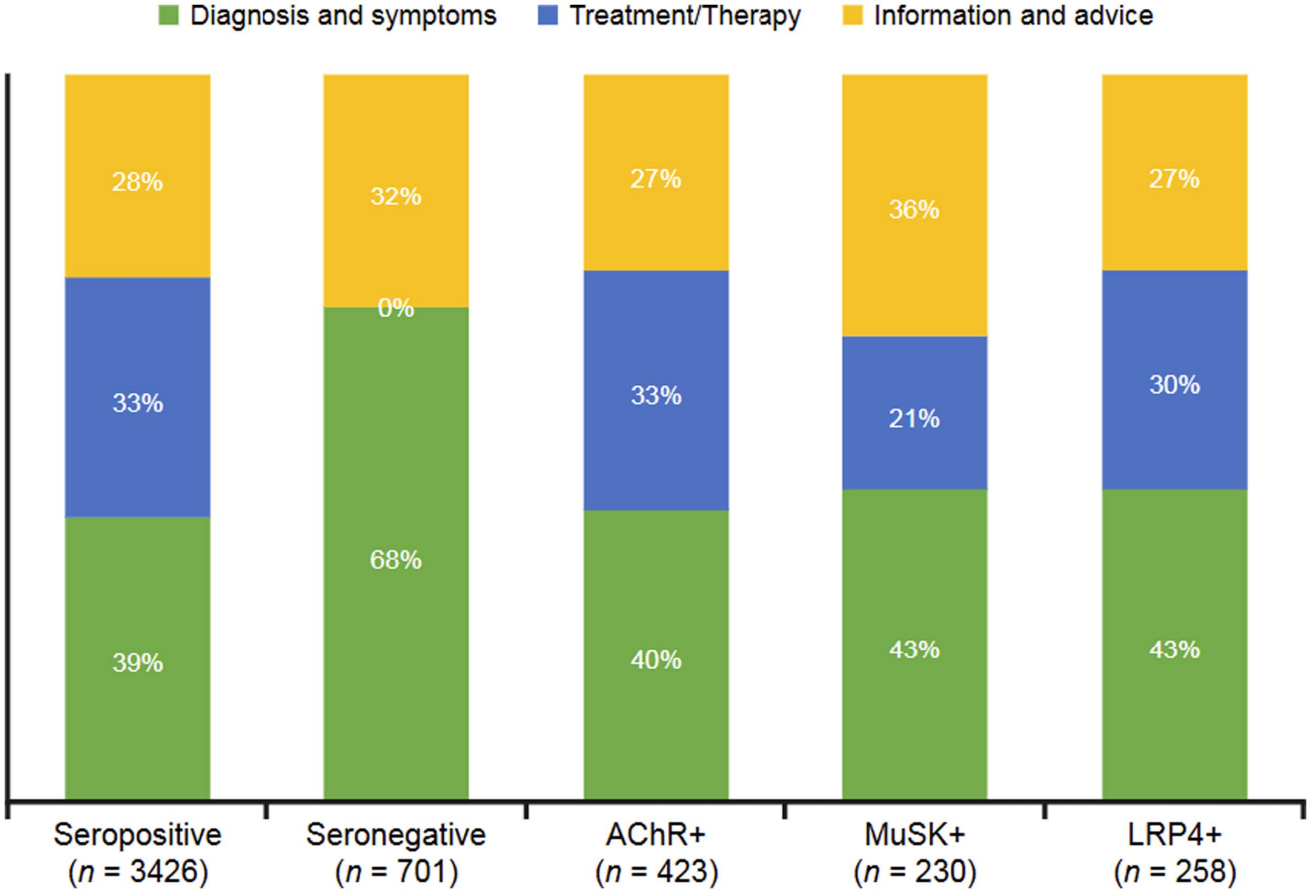
Neutral drivers for sentiments expressed in digital conversations by MG serostatus. The dominant categories of reasons underlying neutral sentiments were diagnosis and symptoms, treatment/therapy, and information and advice. AChR, acetylcholine receptor; LRP4, low-density receptor-related protein 4; MuSK, muscle-specific receptor tyrosine kinase.

Neutral drivers (Figure 4) were characterized as topics related to information seeking. Categories included diagnosis and symptoms, treatment/therapy, and information and advice. Most conversations across all groups were related to seeking information on diagnosis and symptoms (39–68% across all groups). Seronegative conversations overwhelmingly focused on diagnosis and symptoms; MuSK+ conversations were interested in information and advice. AChR+ and LRP4 + subgroups were also likely to seek information on treatment/therapy (33 and 30%, respectively).

**Figure 5:**
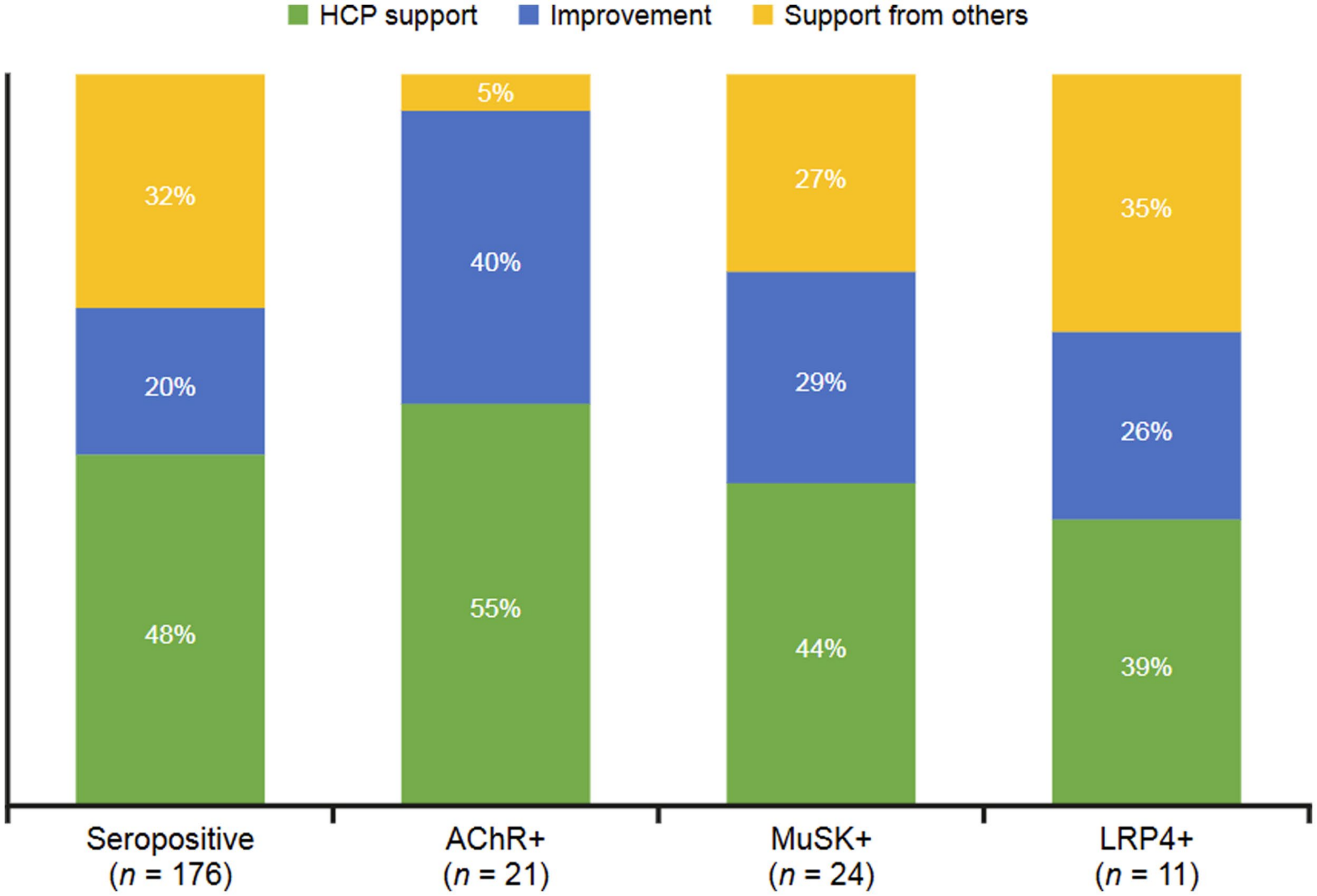
Positive drivers for sentiments expressed in digital conversations by MG serostatus. The dominant categories of reasons underlying positive sentiments were HCP support (receiving information from trusted HCPs), improvement (alleviating symptoms and increasing comfort), and support from others (alleviating loneliness and isolation). AChR, acetylcholine receptor; HCP, healthcare professional; LRP4, low-density receptor-related protein 4; MuSK, muscle-specific receptor tyrosine kinase.

Although positive conversations about MG were rare in this population, thematic groupings were gathered (Figure 5). These drivers included HCP support (receiving information from HCPs they trust), improvement (alleviating symptoms and increasing comfort), and support from others (alleviating loneliness and isolation). Seropositive (48%), AChR+ (55%), MuSK+ (44%), and LRP4 + (39%) conversations identified HCP support as the predominant driver of positive feelings. No positive drivers were identified among seronegative conversations.

### Topics and mindsets

**Figure 6:**
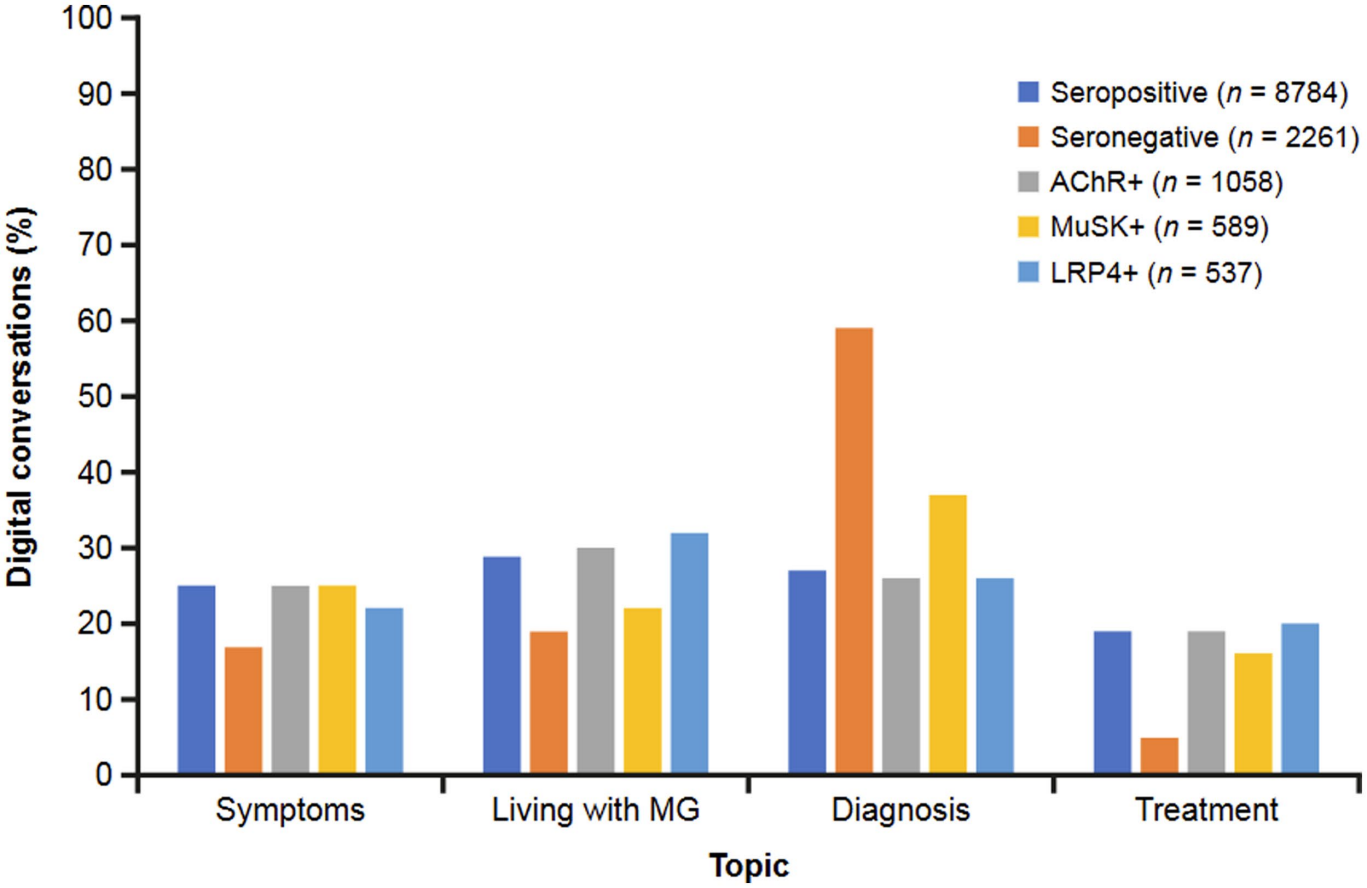
Topics discussed in digital conversations by MG serostatus. The predominant topics discussed in digital conversations were symptoms, living with MG, diagnosis, and treatment. AChR, acetylcholine receptor; LRP4, low-density receptor-related protein 4; MuSK, muscle-specific receptor tyrosine kinase.

The MG-related topics differed to some extent by serostatus (Figure 6). Seropositive conversations were focused on the experience of living with MG (29%), while seronegative conversations were predominantly focused on diagnosis (59%). The seropositive group discussed MG treatment in 19% of conversations, while only 5% of conversations among seronegative were focused on treatments. AChR+ conversations focused on living with MG (30%), and MuSK+ conversations predominantly focused on MG diagnosis (37%), followed by symptoms (25%). The LRP4 + subgroup mostly discussed living with MG (32%) and diagnosis (26%).

**Figure 7:**
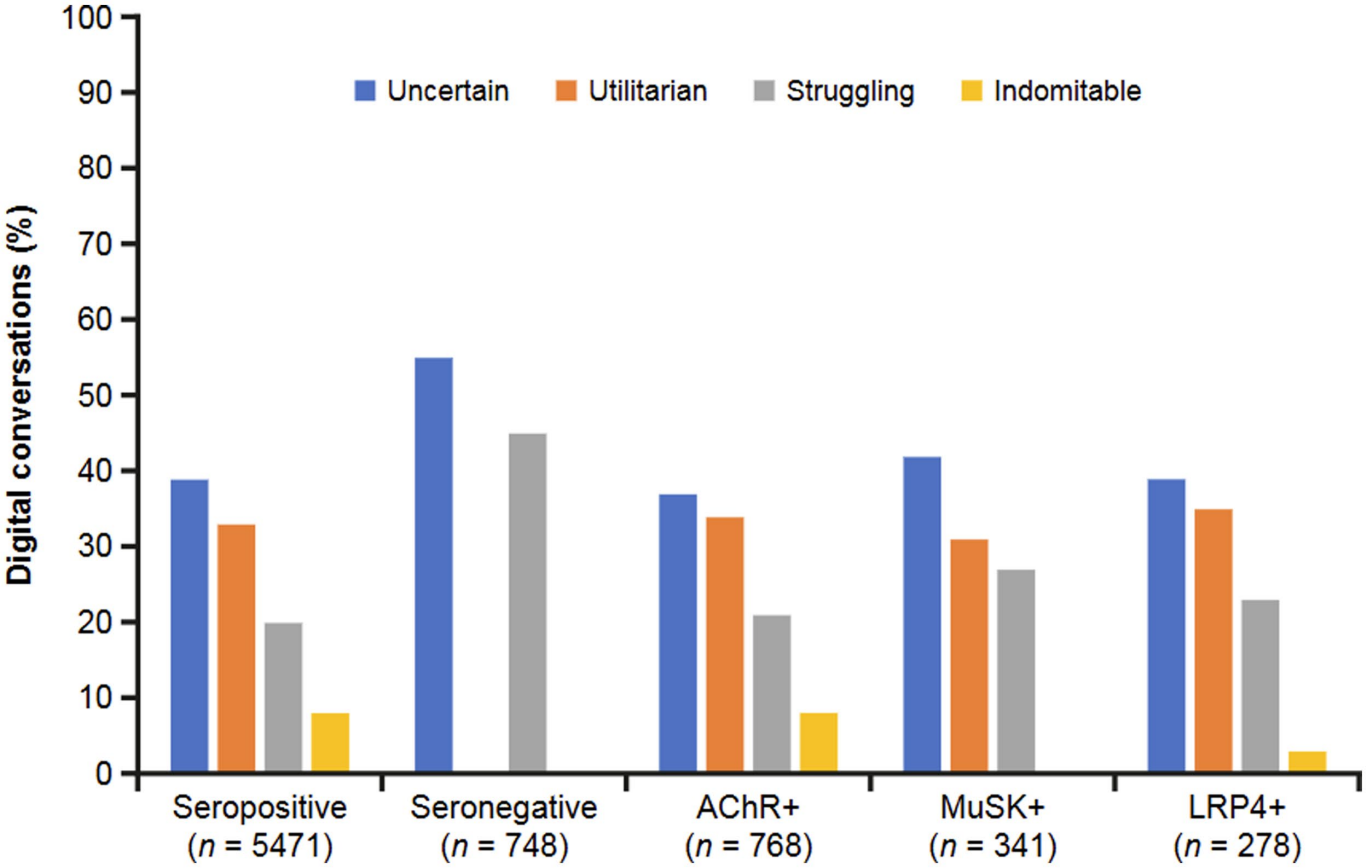
Overarching mindsets described in digital conversations by MG serostatus. Mindsets were defined as uncertain (a sense of unpredictability and insecurity regarding the future, and a lack of confidence in coping with the challenges of MG), utilitarian (a more pragmatic approach focused on factual information), struggling (ongoing difficulty coping with the challenges of MG), and indomitable (a strong and resilient attitude focused on overcoming obstacles, adaptation, and having an optimistic perspective on the challenges of MG). AChR, acetylcholine receptor; LRP4, low-density receptor-related protein 4; MG, myasthenia gravis; MuSK, muscle-specific receptor tyrosine kinase.

Thematic analysis of patient and/or caregiver conversations led to the creation of four primary categories to reflect the mindset of the individual posting (Figure 7). An uncertain mindset dominated across all groups, ranging from 37% to 55%, with seronegative conversations at the highest end of the range. A utilitarian mindset was evident in the overall seropositive conversations (33%), as well as MuSK+ (31%) and LRP4 + (35%) conversations; however, conversations in this realm were absent among AChR+ and seronegative conversations. Additionally, seronegative conversations commonly showed a struggling mindset (45%). An indomitable mindset was rare across all groups.

### Barriers and drivers to treatment

**Figure 8:**
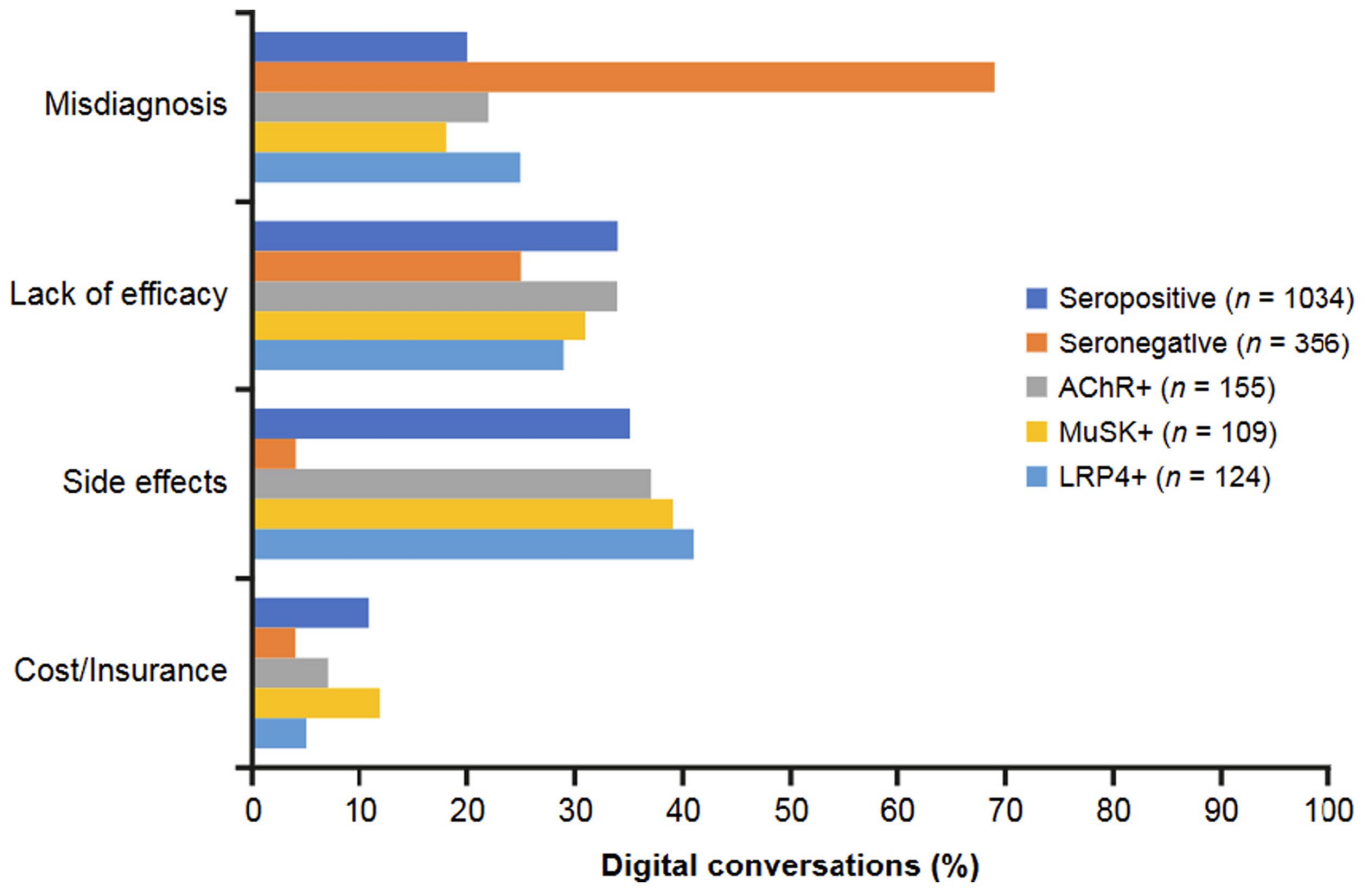
Treatment barriers identified in digital conversations by MG serostatus. Barriers preventing patients from adopting or adhering to treatment included misdiagnosis, lack of treatment efficacy, side effects of treatments, and cost/insurance. AChR, acetylcholine receptor; LRP4, low-density receptor-related protein 4; MuSK, muscle-specific receptor tyrosine kinase.

An examination of the barriers that prevent patients from adopting or adhering to MG treatments (Figure 8) showed that the seropositive group tended to focus on the personal impact of treatment—specifically, side effects (35%) and lack of efficacy (34%). Within this group, the AChR+ subgroup discussed side effects in 37% of conversations. MuSK+ and LRP4 + conversations also mentioned side effects, most often as a barrier to treatment (39 and 42%, respectively). Seronegative conversations focused much more on misdiagnosis as a barrier to treatment (69%), with another quarter of these discussions revealing lack of efficacy as a top barrier. Cost and insurance were least frequently mentioned as barriers across groups; these issues were most frequently discussed in MuSK+ conversations (12%).

**Figure 9:**
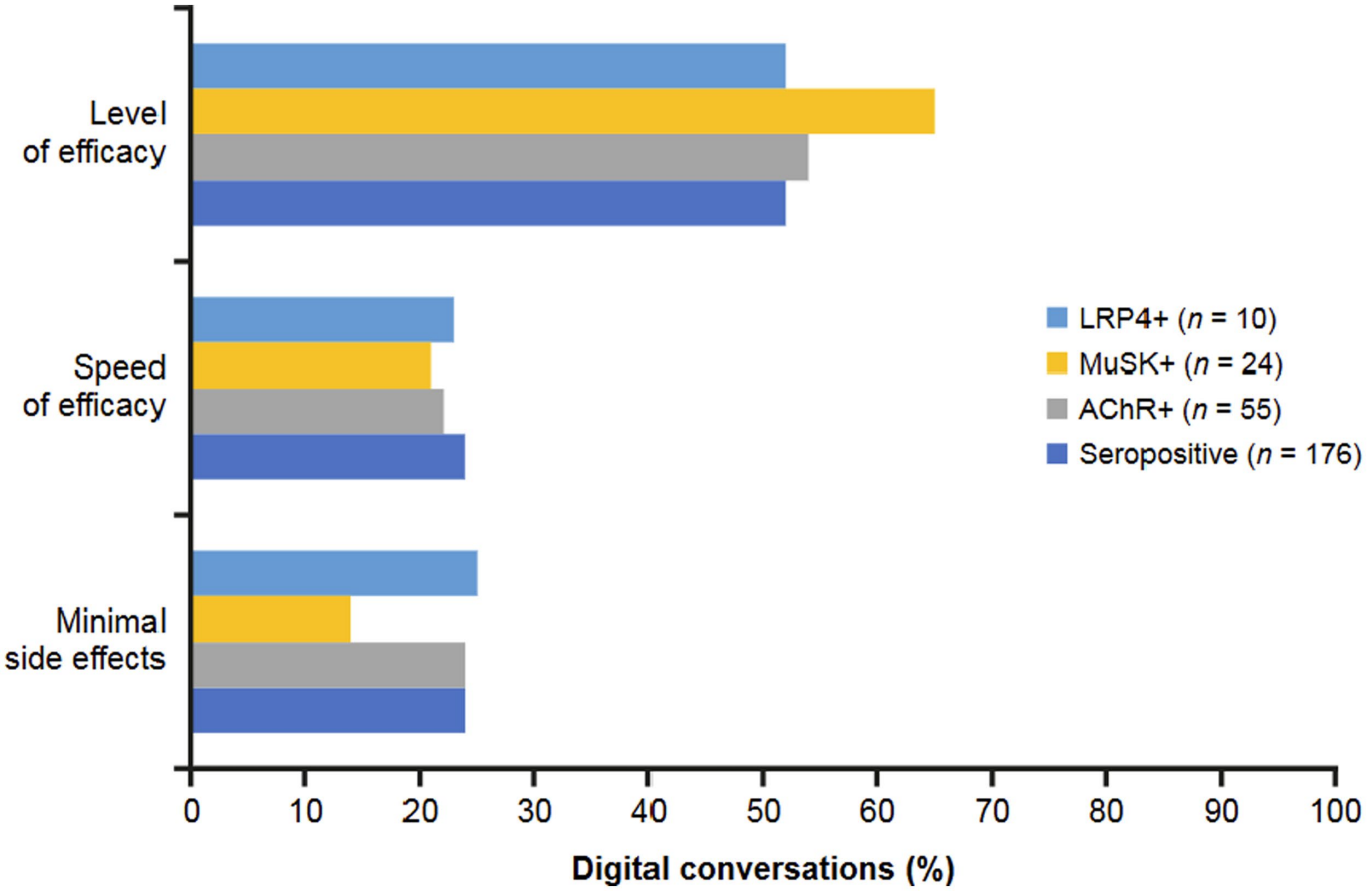
Drivers to treatment identified in digital conversations by MG serostatus. Drivers encouraging patients to adopt or adhere to treatment included minimal side effects (least amount of effect or negative impact), speed of efficacy (feeling quick relief), and level of efficacy (perception of significant improvement in symptoms). Seronegative conversations did not discuss any reasons why patients are driven to adopt or adhere to MG treatment. AChR, acetylcholine receptor; LRP4, low-density receptor-related protein 4; MuSK, muscle-specific receptor tyrosine kinase.

The most frequent drivers of adoption or adherence to treatment that were found among the digital conversations included minimal side effects (least amount of effect or negative impact), speed of efficacy (feeling quick relief), and level of efficacy (perception of significant improvement in symptoms) (Figure 9). The seropositive group most often cited level of efficacy (symptom relief and duration of relief; 52%) as a driver to adopt or adhere to MG treatment. This group was also driven to adopt or adhere to MG treatment by the speed with which treatment begins to work on their symptoms and the ability to experience treatment with minimal side effects in equal measure (24%). AChR+ conversations mirrored overall seropositive treatment drivers with 52% of posts related to level of efficacy, 24% to minimal side effects, and 22% to speed of efficacy. Most MuSK+ conversations were related to level of efficacy (65%), followed by speed of efficacy (21%) and minimal side effects (14%). The LRP4 + subgroup most often cited level of efficacy (52%), followed by minimal side effects (25%) and speed of efficacy (23%). The seronegative group did not discuss any reasons why they are driven to adopt or adhere to MG treatment.

## Discussion

The patient/caregiver voice via digital conversations revealed a high degree of concern across the spectrum of serotypes, specifically related to symptoms, impact on life, misdiagnosis, and treatment. Sentiments, topics, mindsets, and barriers/drivers to treatment differed more profoundly between seropositive and seronegative groups than between different seropositive subtypes. However, regardless of antibody status, negative conversations were dominant overall, potentially indicating a lack of stability in disease management. Positive conversations were rare, sentiments were highly negative, and mindsets toward MG showed a high degree of uncertainty and absence of indomitability, which could have implications for disease management. Misdiagnosis, treatment efficacy, and side effects were frequent topics of conversation, suggesting a lack of satisfaction related to standard-of-care treatments for MG.

Negative drivers of conversations were more common with the seronegative group than the seropositive group. Approximately half of the negative conversations in the seronegative group concerned problems with misdiagnosis, compared with approximately one-quarter of conversations among the seropositive group. Negative conversations focusing on symptom severity were also more common in the seronegative than seropositive group. In contrast, very few negative conversations in the seronegative group discussed treatment issues compared with one-quarter of conversations in the seropositive group. Neutral drivers of conversations, characterized as topics related to information seeking, overwhelmingly concerned diagnosis and symptoms among seronegative conversations, whereas treatment and information/advice topics were also important among seropositive conversations. Likewise, topics related to diagnosis predominated in the seronegative group. Conversations about treatment barriers focused mainly on misdiagnosis in the seronegative group, with lack of efficacy also discussed; very few conversations in the seronegative group considered side effects or cost and insurance issues as barriers. In contrast, conversations about treatment barriers in the seropositive group were more evenly distributed among lack of efficacy, misdiagnosis, and side effects, with cost and insurance issues also discussed. While the seropositive group discussed the level of efficacy of treatments as well as speed of treatment efficacy as drivers for adhering to treatment, the seronegative group did not identify any positive drivers for treatment.

The differences between seronegative and seropositive groups found in our digital conversations study reflect the findings of previous studies reporting that the absence of autoantibodies is associated with significantly longer delays in MG diagnosis and treatment for seronegative patients (5, 15). Timely diagnosis of MG and identification of serologic subtype is critical to effectively manage the disease, improve patient quality of life, and limit additional healthcare resource use (16). Much less is known regarding clinical presentation and efficacy of treatment among seronegative patients, in part due to low representation in clinical trials (17), as well as the lack of modern treatments for these patients (12).

In the seropositive group, negative conversations concerning misdiagnosis were more common with the MuSK+ or LRP4 + subgroup than with the AChR+ subgroup. Negative conversations about symptom severity were less common with MuSK+. Compared with the AChR+ and LRP4 + subgroups, neutral conversations in the MuSK+ subgroup focused less on treatment and more on information and advice. In the AChR+ subgroup, positive conversations were predominated by HCP support and improvement (in symptoms), while support from others was also an important theme among MuSK+ and LRP4 + conversations. Diagnosis was the most common topic discussed in the MuSK+ subgroup with less attention paid to symptoms, living with MG, and treatment; there was a more equal distribution of these topics in the AChR+ and LRP4 + subgroups.

Less frequent negative conversations about diagnosis and more frequent positive conversations about HCP support and symptom improvement in the AChR+ subgroup likely reflects that these patients/caregivers comprise by far the highest proportion of patients with MG and have the most treatment options available to them, including more modern treatments (2, 7, 8). MuSK+ and LRP4 + MG subtypes are uncommon and result in particular challenges. This may, in part, explain the higher frequency of conversations focused on diagnosis and misdiagnosis among these subtypes in our study. Minimizing diagnostic delay is crucial for the appropriate treatment and management of patients with the MuSK+ subtype. Poor response to traditional treatments and lack of approved modern treatments might also have driven the higher proportion of information-seeking conversations in the MuSK+ subgroup. Conversations about treatment barriers focused frequently on side effects and treatment efficacy among all seropositive subgroups. Cost and insurance were more commonly cited as a treatment barrier in MSK + conversations than AChR+ or LRP4 + conversations. Level of efficacy was the most important driver to treatment for all seropositive subgroups, with speed of efficacy and side effects being discussed less frequently.

All patients with MG face clinical challenges in achieving symptom control using tolerable options, as highlighted by one patient in our study who described how they felt that their treatment “hadn’t helped at all.” Jackson et al. conducted semi-structured interviews of patients with MG and reported that obtaining symptom stability was a major treatment goal (18). The fluctuating and unpredictable nature of MG symptoms was found to have a substantial impact on all aspects of patients’ lives. Until the recent emergence of biologics and other advanced treatments for MG, standard-of-care therapies relied on acetylcholinesterase inhibitors, corticosteroids, and immunosuppressants. Most of these treatments are effective for many patients with MG; however, 15% of patients are poor or non-responders and many of these therapies are associated with long-term adverse effects (19). A qualitative study in 14 patients with MG found that side effects of MG treatment were of particular concern, especially blood clots, infection/decreased immunity, weight gain, and diarrhea (20). Additional studies with larger patient samples demonstrate that patients’ disease burden and symptoms are not well managed by conventional treatments.

Both seropositive (all subtypes) and seronegative groups felt uncertainty about their MG, which is consistent with research into other rare chronic diseases. For example, like MG, the diagnostic journey for neuromyelitis optica spectrum disorder (NMOSD) is also complicated by significant variability in clinical signs and disease progression, and it is frequently misdiagnosed, particularly in patients initially seronegative for established biomarkers (21). A recent global interview-based study showed that approximately 25% of patients with seropositive NMOSD were first misdiagnosed with conditions such as multiple sclerosis, idiopathic myelitis, optic neuritis, and stroke (22). Similar to the rate of misdiagnosis in our study across serotypes and inherent to diagnoses based on the exclusion of other conditions, this may cause increased uncertainty for patients throughout their diagnostic journey.

Limited research has been conducted examining the impact of MG from the patient perspective, particularly as it pertains to the various serological subtypes. Consistent with our findings of negative sentiments and mindsets, in the multi-country MyReal-World MG survey, approximately half of respondents reported anxiety and approximately one-third reported an impact on work or study (23). Small, qualitative studies have provided valuable insights into the MG patient experience in dealing with the challenges of diagnosis, treatment, and symptom management (18, 24). Results from elicitation interviews of 28 patients highlighted the physical and emotional impact of MG (18). Patients expressed frustration around unpredictable fluctuations in symptoms relating to vision, breathing, fatigue, and swallowing, which impacted on their emotional, social, and economic well-being. In a qualitative analysis of 54 patients with MG, patients primarily expressed themes around symptom fluctuation, as well as treatment issues, a lack of connection to HCPs, and overall mental health concerns (24). Similar to our study, two analyses of digital conversations among patients with MG reported a high degree of negative sentiment (25, 26). Negative conversations mainly featured discussions on the impact on life of MG, misdiagnosis, treatment issues, and symptoms (25, 26). In focus groups, participants prioritized convenient treatments that effectively managed their symptoms with minimal side effects (26).

Among serostatus and subtype groups, we have identified nuanced differences in issues including impact of symptoms, diagnosis and treatment, and relationships with HCPs. Social media, such as the outlets analyzed in the current study, can be an important source of support for patients and caregivers to obtain disease knowledge, express feelings, and create a sense of community (27). The paucity of conversations exemplifying an indomitable mindset reflects a lack of hope or sense of control over the disease. These findings underscore the unmet need for treatments that better control symptoms and reduce MG exacerbations or crises, which can improve patients’ lives and provide hope for those living with MG.

## Strengths and limitations

A strength of this study is in the unique application of advanced search and online data extraction technology to further our understanding of the experiences of patients with MG and their caregivers. We have used “big data” to analyze unprovoked patient and caregiver conversations. Because MG is a rare disease, patients within certain MG subtypes tend to lack representation in both clinical and observational research due to their disproportionately low numbers within the overall MG population. For this reason, the patient-inclusive perception of their current disease state held value to warrant the risks associated with including self-reported information. It is critical that their perspectives and experiences are collected and analyzed to improve their MG experience and prognosis. Additional strengths include the large sample of conversations examining the patient with MG and caregiver perspectives. Our results are further strengthened by being truly patient and caregiver driven, as conversations were unsolicited by researchers or HCPs.

Limitations of the study included only collecting US conversations and only examining conversations where serostatus and subtype could be identified. The analysis was conducted on posted conversations rather than individuals and was subjective in the way the conversations were categorized. Additionally, we were unable to verify that the conversations were posted by or about someone with an official diagnosis of MG, nor could we verify serological status. It is noteworthy that there were approximately half as many conversations identified as MuSK+ or LRP4 + than conversations identified as AChR+, given that the AChR+ serotype represents most seropositive cases (2–4). The distribution of serotypes among conversations therefore does not represent the overall population of patients with MG. Moreover, given that health-related social media users are more likely to be younger or women (28), digital conversation data have several inherent biases, including potentially skewing toward those populations. In addition, patients might be more likely to focus on the topics of diagnosis, treatment, and quality of life over other MG-related topics. The individuals who discuss MG on social media might not, therefore, reflect the overall population with MG, which could limit the generalizability of the findings. Although duplicate posts were removed, individuals might have engaged in multiple conversations, either within the same platform or on different platforms; the views of the same individual could therefore be captured several times. Additionally, generalizing to larger seropositive, seronegative, AChR+, MuSK+, and LRP4 + patients and their caregivers should be done with caution, as many factors influence online participation.

## Conclusion

This analysis presents the varying perspectives of patients with MG and their caregivers, and the similarities and differences in sentiments, barriers, and drivers by serostatus. Notably, there was a lack of conversations with positive sentiment across all groups. Misdiagnosis dominated posts among the seronegative group, highlighting opportunities for research into utility and access to serologic assays to improve diagnosis and subsequent care in this population. Treatment-related concerns were common for all groups, calling for greater use of effective and tolerable treatments. An uncertain mindset dominated regardless of serotype, which suggests potential opportunities to improve HCP and patient communication and information sharing through avenues such as accessible educational materials and shared decision-making.

## Data Availability

The data that support the findings of this study are available from the authors upon reasonable request and with permission of CulturIntel DBA Human Dot Plus.

## References

[1] Beloor Suresh A Asuncion RMD. Myasthenia gravis. Treasure Island, FL: StatPearls Publishing LLC (2024). 32644757

[2] Silvestri NJ Narayanaswami P. The diagnosis of myasthenia gravis: the sensitive issue of specificity. Muscle Nerve. (2023) 67:436–8. doi: 10.1002/mus.27814, PMID: 36897276

[3] Dresser L Wlodarski R Rezania K Soliven B. Myasthenia gravis: epidemiology, pathophysiology and clinical manifestations. J Clin Med. (2021) 10:2235. doi: 10.3390/jcm10112235, PMID: 34064035 PMC8196750

[4] Gilhus NE Verschuuren JJ. Myasthenia gravis: subgroup classification and therapeutic strategies. Lancet Neurol. (2015) 14:1023–36. doi: 10.1016/S1474-4422(15)00145-3, PMID: 26376969

[5] Leite MI Jacob S Viegas S Cossins J Clover L Morgan BP. IgG1 antibodies to acetylcholine receptors in 'seronegative' myasthenia gravis. Brain. (2008) 131:1940–52. doi: 10.1093/brain/awn092, PMID: 18515870 PMC2442426

[6] Devic P Petiot P Simonet T Stojkovic T Delmont E Franques J. Antibodies to clustered acetylcholine receptor: expanding the phenotype. Eur J Neurol. (2014) 21:130–4. doi: 10.1111/ene.12270, PMID: 24112557

[7] Sanders DB Wolfe GI Benatar M Evoli A Gilhus NE Illa I. International consensus guidance for management of myasthenia gravis. Executive summary. Neurology. (2016) 87:419–25. doi: 10.1212/WNL.0000000000002790, PMID: 27358333 PMC4977114

[8] Narayanaswami P Sanders DB Wolfe G Benatar M Cea G Evoli A. International consensus guidance for management of myasthenia gravis. 2020 update. Neurology. (2021) 96:114–22. doi: 10.1212/WNL.0000000000011124, PMID: 33144515 PMC7884987

[9] Howard JF Bril V Vu T Karam C Peric S Margania T. Safety, efficacy, and tolerability of efgartigimod in patients with generalised myasthenia gravis (ADAPT): a multicentre, randomised, placebo-controlled, phase 3 trial. Lancet Neurol. (2021) 20:526–36. doi: 10.1016/S1474-4422(21)00159-9, PMID: 34146511

[10] Bril V Drużdż A Grosskreutz J Habib AA Mantegazza R Sacconi S. Safety and efficacy of rozanolixizumab in patients with generalised myasthenia gravis (MycarinG): a randomised, double-blind, placebo-controlled, adaptive phase 3 study. Lancet Neurol. (2023) 22:383–94. doi: 10.1016/S1474-4422(23)00077-7, PMID: 37059507

[11] Sansoni J Menon N Viali L White S Vucic S. Clinical features, treatments, their impact, and quality of life for myasthenia gravis patients in Australia. J Clin Neurosci. (2023) 118:16–22. doi: 10.1016/j.jocn.2023.09.023, PMID: 37844489

[12] Myasthenia Gravis Foundation of America. *How is seronegative myasthenia gravis diagnosed?* (2025). Available online at: https://myasthenia.org/Understanding-MG/Seronegative-MG-Resource-Center#:~:text=Diagnosing%20seronegative%20myasthenia%20gravis%20can,some%20treatments%20are%20antibody%20specific (Accessed January 14, 2025).

[13] Castilla-Puentes R Dagar A Villanueva D Jimenez-Parrado L Valleta LG Falcone T. Digital conversations about depression among Hispanics and non-Hispanics in the USA: a big-data, machine learning analysis identifies specific characteristics of depression narratives in Hispanics. Ann General Psychiatry. (2021) 20:50. doi: 10.1186/s12991-021-00372-0, PMID: 34844618 PMC8630887

[14] Son Y Kim C Lee J. FED: fast and efficient dataset deduplication framework with GPU acceleration. arXiv. (2025) 2025:11. doi: 10.48550/arXiv.2501.01046

[15] Vinciguerra C Bevilacqua L Lupica A Ginanneschi F Piscosquito G Rini N. Diagnosis and management of seronegative myasthenia gravis: lights and shadows. Brain Sci. (2023) 13:1286. doi: 10.3390/brainsci13091286, PMID: 37759888 PMC10526522

[16] Evoli A Alboini PE Damato V Iorio R Provenzano C Bartoccioni E. Myasthenia gravis with antibodies to MuSK: an update. Ann N Y Acad Sci. (2018) 1412:82–9. doi: 10.1111/nyas.13518, PMID: 29266255

[17] Martinez-Harms R Barnett C Alcantara M Bril V. Clinical characteristics and treatment outcomes in patients with double-seronegative myasthenia gravis. Eur J Neurol. (2024) 31:e16022. doi: 10.1111/ene.16022, PMID: 37531447 PMC11235949

[18] Jackson K Parthan A Lauher-Charest M Broderick L Law N Barnett C. Understanding the symptom burden and impact of myasthenia gravis from the patient's perspective: a qualitative study. Neurol Ther. (2023) 12:107–28. doi: 10.1007/s40120-022-00408-x, PMID: 36322146 PMC9837342

[19] Iorio R. Myasthenia gravis: the changing treatment landscape in the era of molecular therapies. Nat Rev Neurol. (2024) 20:84–98. doi: 10.1038/s41582-023-00916-w, PMID: 38191918

[20] Bacci ED Coyne KS Poon JL Harris L Boscoe AN. Understanding side effects of therapy for myasthenia gravis and their impact on daily life. BMC Neurol. (2019) 19:335. doi: 10.1186/s12883-019-1573-2, PMID: 31864345 PMC6925439

[21] Delgado-Garcia G Lapidus S Talero R Levy M. The patient journey with NMOSD: from initial diagnosis to chronic condition. Front Neurol. (2022) 13:966428. doi: 10.3389/fneur.2022.966428, PMID: 36147040 PMC9488131

[22] Capobianco M Ringelstein M Welsh C Lobo P deFiebre G Lana-Peixoto M. Characterization of disease severity and stability in NMOSD: a global clinical record review with patient interviews. Neurol Ther. (2023) 12:635–50. doi: 10.1007/s40120-022-00432-x, PMID: 36826457 PMC10043113

[23] Berrih-Aknin S Palace J Meisel A Claeys KG Muppidi S Saccà F. Patient-reported impact of myasthenia gravis in the real world: findings from a digital observational survey-based study (MyRealWorld MG). BMJ Open. (2023) 13:e068104. doi: 10.1136/bmjopen-2022-068104, PMID: 37169499 PMC10186408

[24] Law N Davio K Blunck M Lobban D Seddik K. The lived experience of myasthenia gravis: a patient-led analysis. Neurol Ther. (2021) 10:1103–25. doi: 10.1007/s40120-021-00285-w, PMID: 34687427 PMC8540870

[25] Anderson A Pesa J Choudhry Z Brethenoux C Furey P Jackson L. Patient perceptions of disease burden and treatment of myasthenia gravis based on sentiment analysis of digital conversations. Sci Rep. (2024) 14:7271. doi: 10.1038/s41598-024-57825-1, PMID: 38538905 PMC10973330

[26] Yung M Narayanaswami P Pesa J Choudhry Z Jackson L Deering KL. Patient and care partner perspectives and preferences related to myasthenia gravis treatment: a qualitative study. Health Sci Rep. (2024) 7:e70081. doi: 10.1002/hsr2.70081, PMID: 39323457 PMC11422664

[27] De Martino I D’Apolito R McLawhorn AS Fehring KA Sculco PK Gasparini G. Social media for patients: benefits and drawbacks. Curr Rev Musculoskelet Med. (2017) 10:141–5. doi: 10.1007/s12178-017-9394-7 28110391 PMC5344865

[28] Sadah SA Shahbazi M Wiley MT Hristidis V. A study of the demographics of web-based health-related social media users. J Med Internet Res. (2015) 17:e194. doi: 10.2196/jmir.4308, PMID: 26250986 PMC4705027

